# 24(S)-Hydroxycholesterol protects the *ex vivo* rat retina from injury by elevated hydrostatic pressure

**DOI:** 10.1038/srep33886

**Published:** 2016-09-22

**Authors:** Makoto Ishikawa, Takeshi Yoshitomi, Charles F. Zorumski, Yukitoshi Izumi

**Affiliations:** 1 Department of Ophthalmology, Akita University Graduate School of Medicine, Akita 010-8543, Japan; 2 The Taylor Family Institute for Innovative Psychiatric Research, Washington University School of Medicine, St. Louis, M.O., USA; 3 Center for Brain Research in Mood Disorders, Washington University School of Medicine, St. Louis, M.O., USA; 4 Department of Psychiatry, Washington University School of Medicine, St. Louis, M.O., USA

## Abstract

In the central nervous system, 24(S)-hydroxycholesterol (24(S)-HC) is an oxysterol synthesized from cholesterol by cholesterol 24-hydroxylase (CYP46A1) encoded by the *cyp46a1* gene. In the present study using a rat *ex vivo* glaucoma model, we found that retinal 24(S)-HC synthesis is facilitated by pressure elevation. Moreover, we found that 24(S)-HC is neuroprotective against pressure mediated retinal degeneration. Quantitative real-time RT-PCR, ELISA, and immunohistochemistry revealed that elevated pressure facilitated the expression of *cyp46a1* and CYP46A1. Immunohistochemically, the enhanced expression of CYP46A1 was mainly observed in retinal ganglion cells (RGC). LC-MS/MS revealed that 24(S)-HC levels increased in a pressure-dependent manner. Axonal injury and apoptotic RGC death induced by 75 mmHg high pressure was ameliorated by exogenously administered 1 μM 24(S)-HC. In contrast, voriconazole, a CYP46A1 inhibitor, was severely toxic even at normobaric pressure. Under normobaric conditions, 30 μM 24(S)-HC was required to prevent the voriconazole-mediated retinal damage. Taken together, our findings indicate that 24(S)-HC is facilitated by elevated pressure and plays a neuroprotective role under glaucomatous conditions, while voriconazole, an antifungal drug, is retinotoxic. 24(S)-HC and related compounds may serve as potential therapeutic targets for protecting glaucomatous eyes from pressure-induced injuries.

Glaucoma is a leading cause of irreversible blindness^1^,^2^. The global prevalence of glaucoma for individuals aged 40–80 years is estimated as 3.54%^1^,^2^,^3^,^4^. Among subtypes of glaucoma, acute angle closure attacks (AACA) are ophthalmic emergencies^2^,^5^ characterized by sudden increases in intraocular pressure (IOP) that can reach 80 mmHg^6^, and result in permanent damage to retinal ganglion cells (RGC) within days^5^,^7^. However, the pathogenesis underlying pressure-induced RGC death in AACA remains unclear.

Excitotoxicity is a form of neuronal degeneration induced by sustained activation of glutamate receptors^8^ that is associated with retinal ischemia and thought to contribute to glaucomatous RGC damage^9^,^10^,^11^. Thus, it is hypothesized that functional impairment of glutamate transporters may contribute to the pathogenesis of glaucoma. In support of this, glutamate transporter-knock-out mice show glaucomatous-type damage of the optic nerve^12^ and our prior studies using an *ex vivo* acute glaucoma model^13^,^14^ revealed that elevated hydrostatic pressure inhibited glutamate clearance by the glial glutamate transporter GLAST and the glutamate metabolizing enzyme glutamine synthetase.

Recently, it has been reported that cholesterol metabolism is altered in rat hippocampal models of glutamate-mediated excitotoxicity^15^. The major mechanism of cholesterol removal from the brain is its conversion into 24S-hydroxycholesterol (24(S)-HC), which diffuses out of the neurons and crosses the blood–brain barrier^16^. This reaction is catalyzed by a cytochrome P450 enzyme, cholesterol 24-hydroxylase (CYP46A1), coded by the gene *cyp46a*^17^,^18^,^19^.

As in the brain, cholesterol is synthesized endogenously in the retina^20^, although cholesterol from the systemic circulation can also cross the retinal pigment epithelium and enter the retina^21^. In the retina, CYP46A1 is selectively expressed in the inner retina, particularly in RGC^22^,^23^,^24^. The localization of CYP46A1 in RGC supports a possible role of 24(S)-HC in glaucoma because RGC are the cells most affected by elevated retinal pressure. Consistent with this, Fourgeux *et al*.^25^,^26^ reported that a single nucleotide polymorphism, rs754203, in the *cyp46a1* gene is associated with risk for POAG, though this was not replicated in a subsequent study^27^. Thus, the relationship between *cyp46a1* and glaucoma remains uncertain. Despite this, it is possible that 24(S)-HC synthesis is involved in glaucomatous retinal degeneration, and experimental IOP elevation in rats facilitates retinal *cyp46a1* expression followed by sustained increases in 24(S)-HC^28^. In the present study using an *ex vivo* glaucoma model (Fig. 1A), we examined whether retinal 24(S)-HC synthesis is altered by activation of CYP46A1. We also determined whether 24(S)-HC plays a role in regulating pressure-induced damage.

## Results

### Effects of pressure loading on expression of cholesterol 24-hydroxylase

**Figure 1:**
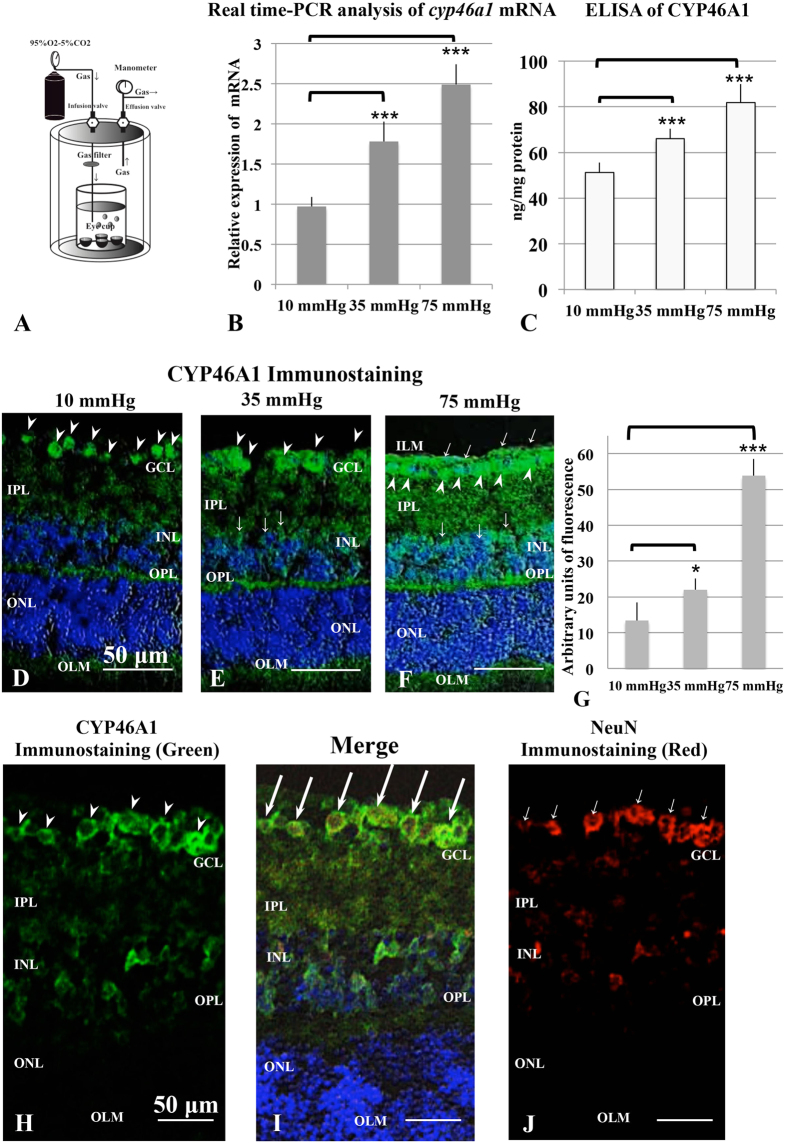
A closed pressure-loading system (**A**) and analysis of cholesterol 24-hydroxylase expression using quantitative real-time RT–PCR, ELISA, and immunohistochemistry (**B–J**). (**A**) The diagram shows the closed pressure-loading chamber. (**B**) Real-time RT-PCR analysis of *cyp46a1* gene mRNA. *Cyp46a1* mRNA expression increased in a pressure-dependent manner and was significantly up-regulated at 35 mmHg and 75 mmHg compared to the control pressure (10 mmHg). ***p < 0.001. (**C**) Protein levels of CYP46A1 in pressure-loaded retinas were measured with ELISA (N = 8 at each experiment). The levels significantly increased at 35 mmHg and 75 mmHg compared to control pressure (10 mmHg). ***p < 0.001. (**D**–**F**) Merge of differential interference contrast images and fluorescence images using DAPI and an antibody against CYP46A1. Panels D–F are at the same magnification. Scale bars, 50 μm. (**D**) Immunostaining was detected in all RGCs (arrowheads) in a retina incubated at 10 mmHg. (**E**) At 35 mmHg, the retina showed more prominent fluorescence in the GCL (arrowheads) and INL (arrows). Weak fluorescence was present in the IPL and OPL. (**F**) Strong immunofluorescence was observed in the GCL, IPL, INL, and OPL at 75 mmHg. Arrowheads and arrows indicate the positive fluorescence in the GCL and INL, respectively. (**G**) Summary of immunostaining studies shows fluorescence intensity by anti-CYP46A1 antibody as mean ± SEM. Fluorescence intensity significantly increased at 35 mmHg and 75 mmHg compared with 10 mmHg. *p < 0.5, ***p < 0.0001. (**H**–**J**) Cryosections double labeled with anti-CYP46A1 (**H**) antibody and anti-NeuN antibody (the RGC marker) (**J**). Arrowheads indicate CYP46A1-positive RGCs in Fig. H, and arrows indicate NeuN-positive RGCs in Fig. J. Merge image (**I**) revealed that CYP46A1-positive cells in the GCL were RGCs. Double labeled RGCs with both antibodies are indicated by long arrows. Panels H–J are at the same magnification. Scale bars, 50 μm.

In initial experiments, we examined the effects of pressure loading on the expression of cholesterol 24-hydroxylase, the enzyme that converts cholesterol to 24(S)-HC, using a closed pressure chamber (Fig. 1A). We found that *cyp46a1* mRNA expression increased in a pressure-dependent manner (Fig. 1B), with significant increase at 35 mmHg (*p* < 0.0001) and 75 mmHg (*p* < 0.0001) compared to 10 mmHg using quantitative real-time reverse transcription (RT)-polymerase chain reaction (PCR) (see Supporting Data of Fig. 1B and Table S1B-1,S1B-2,S1B-3).

Consistent with changes in mRNA, we also found that pressure loading increased expression of CYP46A1 protein. Using enzyme-linked immunosorbent assay (ELISA) measurements, we observed significant increase in CYP46A1 protein at 35 mmHg (*p* < 0.01) and 75 mmHg (*p* < 0.001) compared to 10 mmHg (Fig. 1C and Table S1C).

We also confirmed the effects of elevated hydrostatic pressure on the oxysterol synthetic enzyme using an antibody against CYP46A1 and immunocytochemistry. As reported by other investigators^22^,^23^,^24^, CYP46A1-positive cells were observed mostly in the ganglion cell layer (GCL) and a few cells in the inner nuclear layer (INL) at a normobaric pressure (Fig. 1D). At 35 mmHg, the fluorescent reaction was increased in the GCL and also apparent in the inner plexiform layer (IPL) and outer plexiform layer (OPL) (Fig. 1E). At 75 mmHg, immunofluorescence was prominent in the GCL and INL (Fig. 1F). Fluorescence intensities in each condition are summarized in Fig. 1G (Table S1G). The cells labelled with CYP46A1 in the GCL were considered to be RGCs because they also expressed NeuN (a RGC specific marker)^29^,^30^,^31^ in double labeling experiments (Fig. 1H–J).

### Effects of pressure loading on endogenous levels of cholesterol and 24(S)-HC

**Figure 2:**
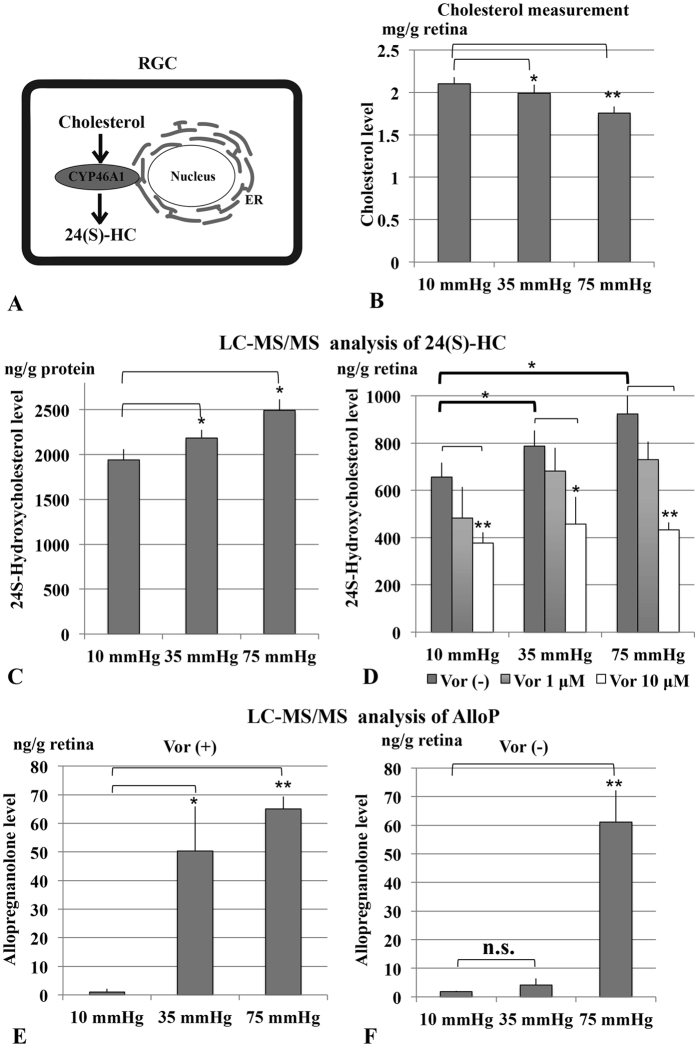
Effects of pressure loading on endogenous levels of cholesterol and 24(S)-HC. (**A**) CYP46A1 catalyzes the conversion of cholesterol to 24(S)-HC in the retina. (**B**) Pressure loading revealed pressure-dependent decreases in retinal cholesterol concentration. *p < 0.05, **p < 0.001. (**C**) LC-MS/MS analysis revealed that 24(S)-HC (ng/g retinal protein) increased in a pressure-dependent manner. *p < 0.05. (**D**) Measurement of 24(S)-HC (per wet retinal weight) in retinal extracts using LC-MS/MS. 24(S)-HC levels significantly increased at 35 mmHg and 75 mmHg compared to control pressure (10 mmHg). Administration of 1 μM voriconazole had no significant effect on 24(S)-HC levels at any pressure, while 10 μM voriconazole significantly depressed 24(S)-HC at each pressure. *p < 0.05, **p < 0.01. (**E**,**F**) Measurement of AlloP (per wet retinal weight) in retinas incubated with (**E**) or without 10 μM voriconazole (**F**) using LC-MS/MS. AlloP levels significantly increased at both 35 mmHg (*p < 0.01) and 75 mmHg (**p < 0.001) compared to control pressure (10 mmHg) in the presence of voriconazole, but only at 75 mmHg in the absence of voriconazole.

Cholesterol hydroxylation is important for the maintenance of cholesterol homeostasis in the retina, where the conversion to 24(S)-HC catalyzed by CYP46A1 represents the major mechanism of cholesterol elimination (Fig. 2A). We thus measured levels of cholesterol (per wet retinal weight), the substrate of CYP46A1, in rat *ex vivo* eyecups, and found a pressure-dependent decrease in cholesterol at 35 mmHg (*p* < 0.05) and 75 mmHg (*p* < 0.001) compared to 10 mmHg (Fig. 2B and Table S2B). Consistently, liquid chromatography-tandem mass spectrometry (LC-MS/MS) analysis revealed that 24(S)-HC (ng/g retinal protein), the product of enzymatic activities of CYP46A1, increased in hyperbaric conditions (p < 0.05 at 35 mmHg and p < 0.05 at 75 mmHg, compared to 10 mmHg) in units of ng/g retinal protein (Fig. 2C and Table S2C) and ng per wet retinal weight (Fig. 2D and Table S2D-1). Administration of 1 μM voriconazole, an inhibitor of CYP46A1^32^ showed no significant influence on 24(S)-HC concentrations, while 10 μM voriconazole significantly diminished 24(S)-HC levels at each pressure (Fig. 2D and Table S2D-2,S2D-3).

Because we previously found that high pressure increases local levels of the neurosteroid, allopregnanolone (AlloP), and AlloP is protective against high pressure^33^, we also examined the effects of pressure on AlloP levels with or without 10 μM voriconazole. Three eyes were examined by LC-MS/MS at each pressure, and AlloP levels are expressed as ng per wet retinal weight (g). In the presence of voriconazole, LC-MS/MS analysis revealed pressure-dependent increases of AlloP at 35 mmHg (*p* < 0.01) and 75 mmHg (*p* < 0.001) compared to 10 mmHg, indicating that the decrement in 24(S)-HC levels with voriconazole has some specificity and does not involve another major endogenous cholesterol-derived product (Fig. 2E and Table S2F). In the absence of voriconazole, AlloP levels at 35 mm Hg did not rise as high as they do in the presence of voriconazole (Fig. 2F and Table S2F). In contrast, the LC-MS/MS analysis revealed a significant increase of AlloP at 75 mm Hg compared to 10 or 35 mm Hg. These results suggest that AlloP production may be saturated by the severe stress of 75 mmHg (so this level does not change with voriconazole), but that the milder stress of 35 mmHg only elevates AlloP levels when cholesterol metabolism to 24(S)-HC is blocked.

### 24(S)-HC preserves retinal histology in the presence of high pressure

**Figure 3:**
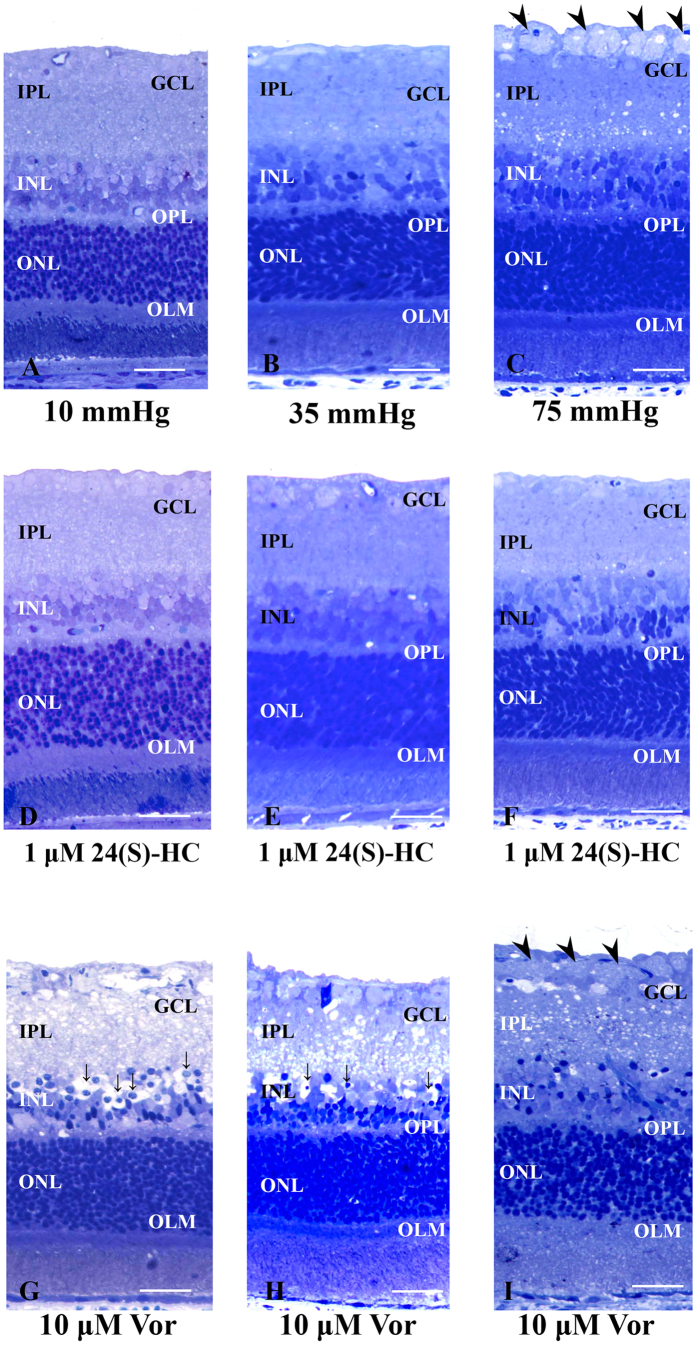
Light micrographs of pressure-dependent changes in the middle part of the retina incubated with 24(S)-HC or 10 μM voriconazole. (**A–C**) Light micrographs of pressure- dependent changes in the middle part of the retina. (**A**,**B**) In retinas incubated at 10 mmHg (**A**) and 35 mmHg (**B**), no abnormal changes were detected in any retinal layers. (**C**) Prominent swelling of optic nerve fibers (arrowheads) was observed in a retina incubated at 75 mmHg. Small vacuoles were also present in the IPL. Retinal degeneration was not observed in other retinal layers. (**D–F**) Light micrographs of the middle part of the retina incubated with 1 μM 24(S)-HC at 10 mmHg (**D**), 35 mmHg (**E**), and 75 mmHg (**F**). Administration of 1 μM 24(S)-HC exhibited no remarkable changes at any pressure. (**G–I**) Light micrographs of the middle part of the retina incubated with 10 μM voriconazole at 10 mmHg (**G**), 35 mmHg (**H**), and 75 mmHg (**I**). Administration of 10 μM voriconazole produced retinal damage characterized by edematous changes in the IPL and bull’s eye formation in the INL (fine arrows) at each pressure. The retinal damage induced by 10 μM voriconazole was most prominent at 10 mmHg. Note the axonal swelling in the NFL (arrowhead) at 75 mmHg (i). Scale bars, 15 μm.

Consistent with our previous reports^13^,^14^, retinas incubated at 10 mmHg (Fig. 3A) or 35 mmHg (Fig. 3B) exhibited no remarkable changes in morphology. However, retinas incubated at 75 mmHg showed axonal swelling in the nerve fiber layer (NFL), though the other retinal layers remained intact except for the INL and IPL where small vacuoles were present (Fig. 3C and Fig. S3-1). In the presence of 1 μM 24(S)-HC administered for the 24 hour incubation period, retinas exhibited no remarkable changes at any pressure (Fig. 3D–F). Importantly, exogenous 24(S)-HC prevented the axonal swelling typically observed at 75 mmHg.

A quantitative assessment of structural changes induced by pressure elevation in the absence and presence of 24(S)-HC is summarized in Table 1 (also see Table 1 Source data). The nerve fiber layer thickness (NFLT), neuronal damage score (NDS), and density of damaged cells in the GCL in retinas incubated at 75 mmHg were significantly increased compared to those in control retinas incubated at 10 mm Hg (*p* < 0.0001). By contrast, administration of 1 μM 24(S)-HC resulted in no significant changes in the NFLT, NDS, or density of damaged cells in the GCL in retinas incubated at 75 mmHg compared to normobaric controls.

### A cholesterol 24-hydroxylase inhibitor is severely retinotoxic at low pressure

In contrast to 24(S)-HC, we found that voriconazole was highly neurotoxic. Although administration of 1 μM voriconazole showed no remarkable changes (Fig. S3-3), administration of 10 μM voriconazole resulted in severe retinal damage characterized by edematous changes in the IPL and bull’s eye cellular formation in the INL at each pressure (Fig. 3G–I). Interestingly, the retinal damage induced by voriconazole was most prominent at 10 mmHg and least prominent at the highest pressure (Fig. 3G). A quantitative assessment of structural changes induced by pressure elevation in the presence of voriconazole is summarized in Table 1 (also see Table 1 Source data). Administration of 10 μM voriconazole produced significant increases in the NDS and density of damaged cells in the GCL compared to the retinas incubated without voriconazole at each pressure.

**Figure 4:**
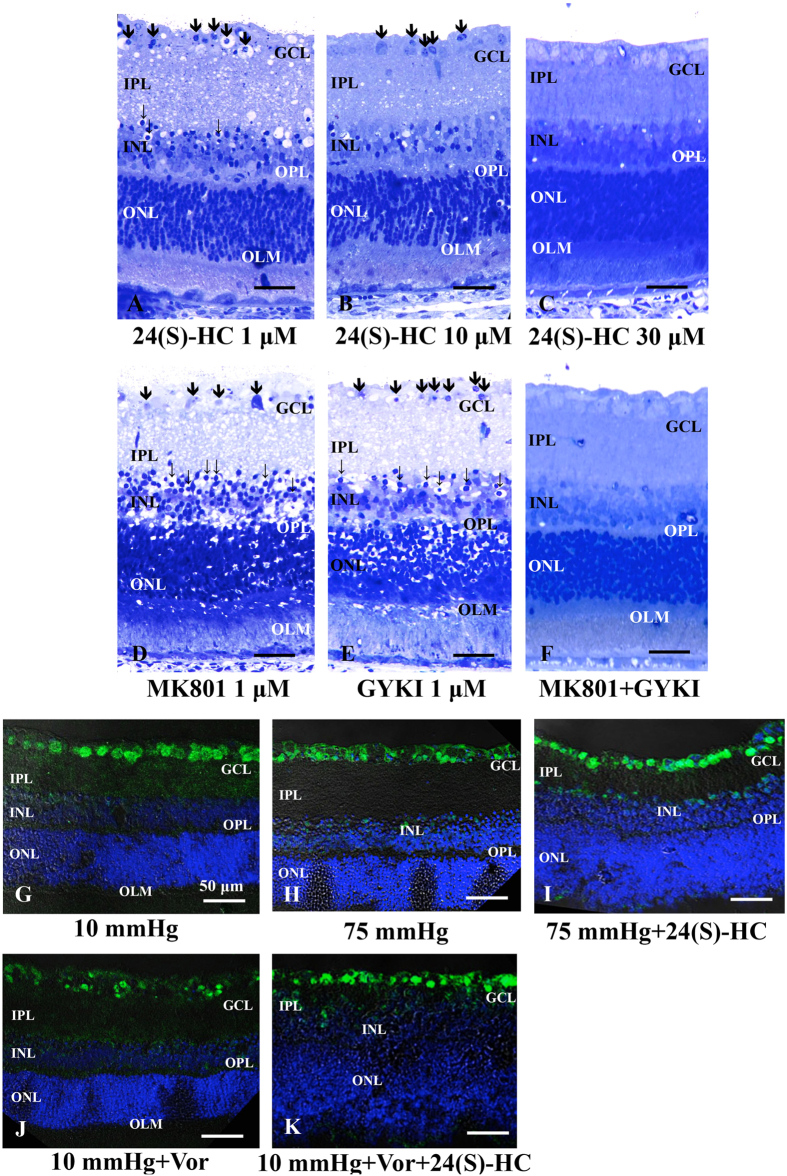
Effects of voriconazole, 24(S)-HC, and glutamate receptor antagonists and RGC survival test using an RGC marker. Light micrographs of the retina incubated with 10 μM voriconazole in combination with 1 μM 24(S)-HC (**A**), 10 μM 24(S)-HC (**B**), or 30 μM 24(S)-HC (**C**) at 10 mmHg. Administration of 1 μM (**A**) or 10 μM 24(S)-HC (**B**) did not completely inhibit the retinal degeneration induced by 10 μM voriconazole. However, 30 μM 24(S)-HC exhibited substantial neuroprotection against voriconazole-induced neuronal damage (**C**). Arrows indicate degenerated cells in the GCL. Note the bull’s eye formation in the INL (fine arrows). (**D–F**) Light micrographs of retinas incubated with 10 μM voriconazole in combination with 1 μM MK801 alone (**D**), 1 μM GYKI alone (**E**), or a combination of 1 μM MK801 and 1 μM GYKI (**F**) at 10 mmHg. (**D**,**E**) Administration of MK801 alone (**D**) or GYKI alone (**E**) did not inhibit the excitotoxic degeneration induced by 10 μM voriconazole at 10 mmHg. Arrows indicate degenerated cells in the GCL. Fine arrows indicate the bull’s eye formation in the INL. (**F**) By contrast, a combination of 1 μM MK801 and 1 μM GYKI exerted almost complete neuroprotection. Scale bars, 15 μm. (**G–K**) Merge of differential interference contrast images and fluorescence images using DAPI and an anti-NeuN antibody. (**G)** At 10 mmHg, anti-NeuN antibody predominantly labeled RGCs. (**H**) Immunofluorescence was reduced in hyperbaric conditions. (**I**) Administration of 24(S)-HC is neuroprotective for RGC survival at 75 mmHg. (**J**) Voriconazole treatment markedly reduced immunofluorescence in the RGCs at 10 mmHg. (**K**) Combination of 30 μM 24(S)-HC and 10 μM voriconazole increased NeuN-positive RGCs. Scale bars, **50 μm**.

In contrast to effects on high pressure alone, we found that retinal degeneration in the presence of 10 μM voriconazole at 10 mmHg was not clearly diminished by 1 μM 24(S)-HC (Fig. 4A). However, higher concentrations of 24(S)-HC (10 and 30 μM) produced concentration-dependent neuroprotection against neuronal damage induced by 10 μM voriconazole (Fig. 4B), with nearly complete protection at 30 μM (Fig. 4C).

A quantitative assessment of structural changes induced by voriconazole and 24(S)-HC at 10 mmHg is summarized in Table 1 (also see Table 1 Source data). Administration of 1 μM 24(S)-HC resulted in no neuroprotection, with increased NDS and density of damaged cells in the GCL in the presence of 10 μM voriconazole at 10 mmHg compared to those in the control retinas incubated without voriconazole at 10 mmHg. At 30 μM, however, co-administration of 24(S)-HC resulted in no significant changes in the NDS and density of damaged cells in the GCL in retinas incubated with 10 μM voriconazole at 10 mmHg compared to control retinas incubated at 10 mmHg in the absence of either agent. An intermediate degree of protection was observed with 10 μM 24(S)-HC. The NFLT did not show significant changes under any of these conditions.

Because voriconazole-induced damage has histological features similar to excitotoxicity, we also examined the effects of glutamate receptor antagonists. In retinas incubated with 10 μM voriconazole at 10 mmHg, administration of either 1 μM dizocilpine (MK801), a NMDA type glutamate receptor antagonist (Fig. 4D), or 1 μM GYKI 52446 (GYKI), an ionotropic non-NMDA-type glutamate receptor antagonist, alone (Fig. 4E) failed to inhibit the retinal damage. However, a combination of MK-801 and GYKI resulted in full neuroprotection in the presence of 10 μM voriconazole (Fig. 4F).

A quantitative assessment of structural changes induced by voriconazole and glutamate receptor antagonists at 10 mmHg is summarized in Table 2 (also see Table 2 Source data). In the presence of either 1 μM MK801 alone or 1 μM GYKI alone, retinas incubated with 10 μM voriconazole at 10 mmHg showed significant increases in the NDS (*p* < 0.0001) and density of damaged cells in the GCL (*p* < 0.0001) compared to control retinas incubated without voriconazole at 10 mmHg. In contrast, the combination of 1 μM MK801 and 1 μM GYKI with voriconazole showed no remarkable changes in the NDS and density of damaged cells in the GCL at 10 mmHg.

### Effects of pressure elevation on RGCs and neuroprotection with 24(S)-HC

RGCs were specifically labeled with anti-NeuN (a RGC nuclear marker) antibody at 10 mmHg (Fig. 4G). Pressure elevation (75 mmHg) decreased the NeuN-positive RGC density in hyperbaric conditions (Fig. 4H), while administration of 1 μM 24(S)-HC reversed high pressure-induced RGC damage at 75 mmHg (Fig. 4I). Administration of 10 μM voriconazole significantly reduced NeuN-positive RGCs at 10 mmHg (Fig. 4J), while co-administration of 24(S)-HC promoted survival of NeuN-positive RGCs in the voriconazole (10 μM) treated retina (Fig. 4K).

**Figure 5:**
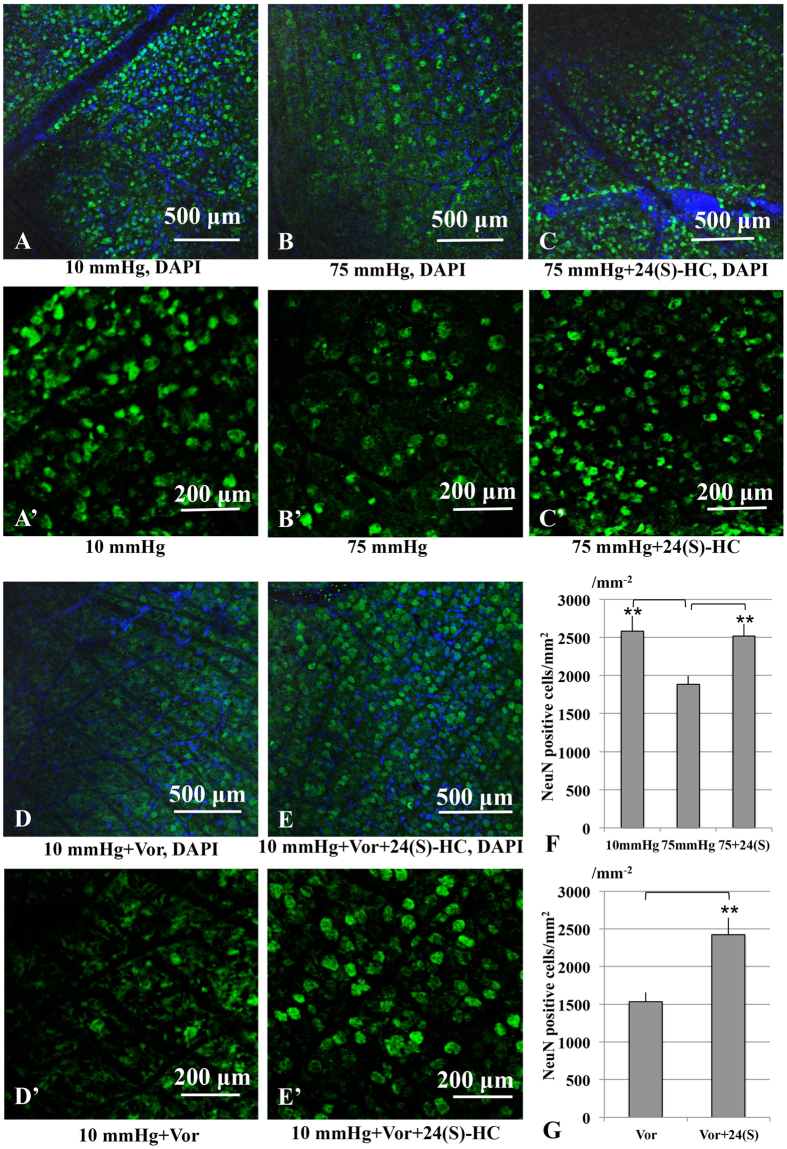
RGC survival test using RGC marker (NeuN) in the whole mount retina. (**A**,**A′**) Confocal images of NeuN-labeled RGCs in a control eye incubated at 10 mmHg. (**B,B′)** Density of NeuN-positive RGCs was reduced at 75 mmHg compared to control pressure (10 mmHg). (**C,C′**) Administration of 24(S)-HC (1 μM) enhanced RGC survival under hyperbaric conditions. (**D,D′**) RGC numbers significantly decreased compared to the control images. (**E,E′)** Administration of 30 μM 24(S)-HC increased the density of NeuN-positive RGCs in the retina treated with 10 μM voriconazole. (**F,G)** The graphs present the number of NeuN-positive cells in the whole mount retina under each experimental condition. **p < 0.01.

In whole mounted retinas, RGC damage induced by pressure elevation was visualized as reduced numbers of cells that were positive for NeuN (Fig. 5A–C). Figure 5A,A′ illustrate examples of confocal images of NeuN-labeled RGCs that were obtained from a control eye incubated at 10 mmHg. Pressure elevation (75 mmHg) reduced the number of cells that were positive for NeuN (Fig. 5B,B′). The confocal images in Fig. 5C,C′ illustrate the neuroprotective effect of 24(S)-HC (1 μM) on RGC survival in hyperbaric conditions. As shown in Fig. 5D,D′, RGC numbers are significantly different from control images consistent with the damage induced by 10 μM voriconazole at 10 mmHg. However, less disruption of the RGCs occurred when 30 μM 24(S)-HC was applied with 10 μM voriconazole (Fig. 5E,E**′**). Figure 5F,G show summaries of RGC survival (also see Table S5F,G).

### Pressure-induced apoptosis and neuroprotection with 24(S)-HC

**Figure 6:**
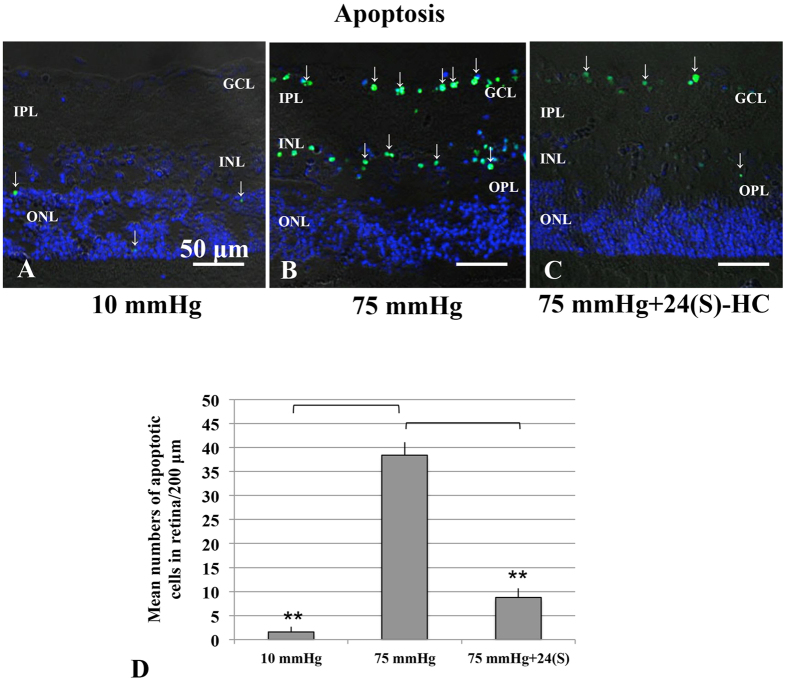
Visualization of apoptotic cells by fluorescence microscopy. **(A–C**) Merge of differential interference contrast images and fluorescence images using DAPI and TUNEL-fluorescent staining of retinas incubated at 10 mmHg (**A**), and 75 mmHg (**C**). (**A**) At 10 mmHg, few TUNEL-positive cells (fine arrows) can be observed within the ONL. (**B**) At 75 mmHg, there was a marked increase of TUNEL-positive cells in the GCL as well as in the INL. (**C)** Administration of 1 μM 24(S)-HC significantly decreased a number of TUNEL-positive cells at 75 mmHg. Scale bars, **50 μm**. **(D)** The graph presents the number of TUNEL-positive RGCs per 200 μm of retinal sections. **p < 0.01.

At 10 mmHg, a small number of TUNEL-positive cells are observed only in the ONL (Fig. 6A). Exposure to elevated pressure induced apoptosis that was apparent in the GCL and to a lesser extent in the INL (Fig. 6B). The number of TUNEL-positive cells was reduced when 1 μM 24(S)-HC was administered (Fig. 6C). The graph in Fig. 6D shows the number of apoptotic cells in the retina in each condition (Table S6D).

## Discussion

24(S)-HC is synthesized from cholesterol by CYP46A1, a neuronal specific enzyme localized to the endoplasmic reticulum^22^. In the rat retina, the enzyme is expressed in the GCL and INL, where the cell bodies of neurons reside, but not in the IPL or OPL that contain axons and synapses^23^. Similar results were shown in mice: the enzyme is expressed in the GCL and partially in the INL^22^. Consistent with these prior studies, we found that the expression of CYP46A1 is exclusively limited to the GCL and INL under normobaric conditions (Fig. 2C). However, in the hyperbaric condition, enzyme expression is not only facilitated, but is also more widely distributed over several retinal layers including the IPL and OPL (Fig. 2D,E), suggesting that the distribution, as well as the strength of the expression, is susceptible to modulation by stressors such as elevated pressure. In the human retina, the distribution of 24-hydroxylase is broader than in rodents^24^.

During pressure elevation in the retina or an AACA *in vivo*, 24(S)-HC levels have been reported to increase. In rats, elevation of intraocular pressure *in vivo* stimulates CYP46A1 within 3 days followed by sustained increases in 24(S)-HC levels^26^. Consistent with this, we observed that expression of the *cyp46a1* gene and CYP46A1 protein (Fig. 2A,B), as well as 24(S)-HC production (Fig. 3A) are enhanced by hyperbaric conditions. Furthermore, we found that enhanced immunofluorescence staining against CYP46A1 is observed over several retinal layers (Fig. 3B,C). In our *ex vivo* glaucoma model, retinas were exposed to hyperbaric conditions for only 24 hours and showed significant elevations in 24(S)-HC levels, suggesting that the induction of the enzyme during AACA can be relatively rapid. As a consequence of up-regulation of CYP46A1, the present study demonstrated a lower concentration of cholesterol and higher concentration of 24(S)-HC in the pressure-loaded retina compared to the retina incubated in the normobaric condition. Based on changes in these metabolite and substrate concentrations, it appears that retinal cholesterol turnover is increased by upregulation of CYP46A1 under hyperbaric conditions.

Is the induction of CYP46A1 during AACA physiological (neuroprotective) or pathological (neurotoxic)? Based on prior studies, it appears that the product of CYP46A1, 24(S)-HC, can have varying effects depending on experimental conditions^34^,^35^,^36^. In the present study, the finding that exogenous administration of 1 μM 24(S)-HC prevents pressure-induced axonal injury and apoptotic RGC death indicates that 24(S)-HC has neuroprotective actions rather than pathological effects. This finding also suggests that facilitated expression of cholesterol 24-hydroxylase triggered by AACA is not a pathological sequela but perhaps a physiological and/or homeostatic mechanism. At this point, however, we do not know whether the preservation of retinal morphology translates into improvement in retinal physiology.

Our results are consistent with a model proposed by Sodero and colleagues^37^,^38^ in which activation of glutamate receptors during stress and aging in the hippocampus promotes translocation of CYP46A1 to the plasma membrane, resulting in cholesterol loss and subsequent stimulation of neuronal survival pathways. Enhanced CYP46A1 expression has also been found to be neuroprotective in *in vivo* models of neurodegenerative illnesses^39^. Together, these findings suggest that decreases in cholesterol via CYP46A1, coupled with increases in levels of 24(S)-HC and perhaps other cholesterol-derived modulators, help to promote neuronal survival under stressful conditions.

To determine whether 24(S)-HC is important for preserving retinal integrity, we also examined the effects of voriconazole. This drug is used clinically as an antifungal agent and inhibits CYP46A1 with relatively high potency^40^. Although the safety of intravitreous injection of voriconazole on retinal function has been described in rabbits^41^, voriconazole transiently impairs bipolar cell function in monkeys^42^ and is associated with retinal dysfunction in rats following repeated systemic administration^43^. Recently, the effects of repeated doses of voriconazole on the vision of healthy human subjects was investigated in a double-blind, placebo-controlled study^44^. This latter study showed that voriconazole reduced scotopic maximal a- and b-wave amplitudes, oscillatory potential amplitude and the 30-Hz photopic flicker response amplitude compared with placebo, while also impairing color vision discrimination. In the present study (Fig. 3G), to our surprise, voriconazole proved to be severely retinotoxic with edematous changes in the IPL and bull’s eye formation in the INL, well-known characteristics of excitotoxic retinal damage^45^,^46^ Indeed, this severe retinal damage was prevented by a combination of antagonists blocking non-NMDA and NMDA ionotropic receptors (Fig. 4F), supporting the idea that the damage involves activation of both types of glutamate receptor. We previously reported that ischemic degeneration of isolated rat retinas is mediated by activation of both types of ionotropic glutamate receptor^47^. Thus, the neurodegeneration induced by voriconazole has histological features that are similar to the damage induced by retinal ischemia. Our findings indicate that voriconazole has significant retinotoxic potential and suggest the need for caution when using the drug under conditions that impair the integrity of the blood-retinal barrier.

Amelioration of voriconazole-induced damage by 24(S)-HC supports the hypothesis that voriconazole induces retinal degeneration via inhibition of CYP46A1. However, contrary to our expectation, full protection against voriconazole required 30 μM 24(S)-HC, a concentration higher than needed to block the effects of high pressure. Importantly, 24(S)-HC is the major brain metabolite of cholesterol and is present at endogenous levels in the tens of micromolar concentration range. Thus, even 30 μM 24(S)-HC is considered to be within the physiological range in human brain homogenates^48^. It may seem odd that 10 μM 24(S)-HC offers only partial protection against voriconazole (Fig. 4A and Table 2), taking into account that 1 μM 24(S)-HC fully prevents axonal swelling at 75 mmHg (Fig. 3F). This finding could reflect the possibility that a reservoir of the oxysterol is depleted by voriconazole because even at 10 mmHg, 24(S)-HC levels are reduced below basal levels by voriconazole (Fig. 2C). Substantial amounts of exogenous 24(S)-HC could thus be needed to replenish basal levels in the presence of voriconazole and diminished endogenous oxysterol production.

The ability of 24(S)-HC to protect retinas against the effects of high pressure and voriconazole is also paradoxical in light of recent studies showing that this oxysterol is a positive allosteric modulator of NMDA receptors in hippocampus, and thus might be expected to worsen excitotoxicity^49^,^50^. Nonetheless, as noted above, prior studies have found that 24(S)-HC can play complex roles under pathological conditions^51^, and another endogenous oxysterol, 25-hydroxycholesterol, is a silent modulator of 24(S)-HC, dampening 24(S)-HC effects on NMDA receptors while having little intrinsic effect on its own^52^. Additionally, cholestane-3β,5α,6β-triol, also an endogenous oxysterol, is a negative allosteric modulator of NMDA receptors, further complicating interpretation of the role of oxysterols under various physiological and pathological conditions^53^. These observations make it important for future studies to determine the various oxysterols that are generated in the retina under different conditions and how these oxysterols interact.

Interestingly, the retinal damage induced by voriconazole was more prominent at 10 mmHg than at 35 or 75 mmHg. This observation cannot be explained by facilitated synthesis of 24(S)-HC at 75 mmHg, because voriconazole inhibits the production of 24(S)-HC at all pressures studied. However, this phenomenon could be explained by the robust rise in AlloP, a GABA-enhancing neurosteroid, at elevated pressures (Fig. 2E,F). We previously reported that exogenously administered AlloP attenuates the development of axonal swelling at high pressures, while blockade of AlloP synthesis at 75 mm Hg results in excitotoxic retinal damage with features akin to voriconazole at low pressure^30^. Based on these findings, it is likely that both 24(S)-HC and AlloP are cholesterol-derived modulators that help to protect the retina endogenously and can potentially be exploited for therapeutic purposes. However, 24(S)-HC appears to be more important for maintaining retinal integrity under normobaric conditions, because at a normobaric pressure blockade of AlloP production does not induce retinal damage^30^, while inhibiting 24(S)-HC synthesis results in severe excitotoxicity (Fig. 3G).

Taken together, our findings indicate that enhanced synthesis of 24(S)-HC has important roles in maintaining retinal integrity under hyperbaric conditions, helping to protect the retina from pressure-induced damage. Thus, 24(S)-HC and related compounds may serve as potential therapeutic targets to protect glaucomatous eyes from pressure-induced injuries.

## Materials and Methods

Protocols for animal use were approved by the Akita Graduate University Animal Studies Committee in accordance with the guidelines of the ARVO Statement for the Use of Animals in Ophthalmic and Vision Research.

### Rat *ex vivo* Eyecup Preparation

Rat *ex vivo* eyecups were prepared from 28–32 day-old male Sprague-Dawley rats (Charles River Laboratories International Inc., Wilmington, MA) as previously described^13^,^14^. The cornea was excised circumferentially with microscissors and the lens and vitreous were removed. The empty eyecup was placed on a flat cutting surface and immersed in ice-cold aCSF. The retina was not detached from the sclera. During experiments, several eyecups (3–8) were placed at the bottom of a 100 ml glass beaker filled with aCSF containing (in mM): 124 NaCl, 5 KCl, 2 MgSO_4_, 2 CaCl_2_, 1.25 NaH_2_PO_4_, 22 NaHCO_3_, and 10 glucose, and incubated at 30 °C for 24 hours using a closed pressure-loading system (Fig. 1A). In this model, the glass beaker was carefully placed at the bottom of an acrylic pressure chamber (2,000 ml) with pH maintained at 7.35 to 7.40. A 95% O_2_-5% CO_2_ gas mixture was delivered via plastic tubing and an air filter (Cat#SLGP033RS, Merck Millipore, Billerica, MA), with the tubing terminating 1 cm above the bottom of the beaker. Gas flow was regulated with an infusion valve and a control dial on the lid of the pressure chamber. The 95% O_2_-5% CO_2_ gas mixture was infused until the pressure reading given by a manometer reached the desired level. The pressure was then locked in place by adjusting the control dial of an effusion valve, and monitored continuously for 24 h. After maintaining the chamber at the set pressure (10, 35, 75 mmHg) for the indicated time, the pressure inside the chamber was carefully decreased by opening the effusion valve. In some experiments, 24(S)-HC (1, 10, 30 μM), voriconazole (1, 10 μM), MK801 (1 μM), and GYKI (1 μM) were added into the aCSF. All chemicals were obtained from Sigma-Aldrich (St. Louis, MO).

### Quantitative real-time RT–PCR

We quantified *cyp46a1* mRNA expression in pressure-loaded eyecup specimens incubated at 10, 35, and 75 mmHg for 24 h based on previously reported methods^14^. At the end of each experiment, the retina of the empty eyecup was detached from sclera and immersed in RNAlater solution (Qiagen, Hilden, Germany). In the present study, six independent experiments were performed for each condition. All PCR reactions were repeated in duplicate, and the average values were used for statistical analysis. The RNA expression levels were normalized to S16 ribosomal protein mRNA (*rps16*) expression (see Supporting data of Fig. 1B. Validation of internal control).

The list of primers used in the present study is summarized as followings;

#### Gene cyp46A1

GenBank accession number NM_001108723.1

Forward (F) and reverse (R) primer sequences

F: GTGCCACCATCGACATCCTG

R: GGTGTTACGGGACGCACTGATAC

Product size (bp) 128

#### Gene rps16

GenBank accession number NM_001169146.1

Forward (F) and reverse (R) primer sequences

F: GAAATGGGCTCATCAAGGTGAA

R: ACGGACCCGGATATCCACA

Product size (bp) 131

The primers were designed using the Perfect Real Time® Support System (Takara). Quantification of the relative expression levels of *cyp46a1* gene was achieved by normalizing to *rps16* using the ΔΔCt method. All data are presented as mean ± SD. Comparisons were performed with Wilcoxon-Mann-Whitney non-parametric test.

### ELISA

After pressure loading, the retina was detached from sclera. Four independent experiments using two retinal samples were performed at each pressure. The retinal samples were rapidly homogenized in phosphate buffered saline (PBS) followed by centrifugation at 4 °C for 15 min at 3,000 g. The supernatants were used to measure the concentrations of cholesterol 24-hydroxylase, using a corresponding ELISA kit (#ABIN2073643 Cytochrome P450, Family 46, Subfamily A, Polypeptide 1 (CYP46A1) ELISA Kit, Antibodies-online.com, Atlanta, GA). According to the manufacturer’s instructions, the absorbance was detected at 450 nm and a standard curve was delineated based on the absorbance of standards. The protein concentration of retinal samples was determined by the Bradford method (Bio-Rad Laboratories, Hercules, CA) using the assay solution and serum-globulin as the standard.

### Immunocytochemistry

For immunocytochemistry, eyecup preparations were fixed with 4% paraformaldehyde-0.1 M phosphate buffer for 2 hours at 4 °C (5 animals per experimental group) at the end of each experiment. Eyecups were washed with ice cold PBS and incubated in blocking solution (1% donkey serum/PBS) for 2 h at 25 °C. Eyecup samples were then embedded in OCT compound (Sakura Global Holdings, Tokyo, Japan), and frozen with liquid nitrogen. Ten μm cryosections were incubated with rabbit anti-CYP46A1 polyclonal antibody (Cat#12486-1-AP, AB_2090661, Proteintech, Rosemont, IL) diluted 1:100 in blocking solution or rabbit anti-NeuN polyclonal antibody (Cat#ab104225, Abcam, Cambridge, MA) (1:200) for 24 h at 4 °C. After incubation with primary antibody, slices were rinsed with PBS and incubated with a secondary antibody, biotinylated goat anti-rabbit immunoglobulin G (IgG) (H + L) (diluted 1:500) (Cat#62-6140, Zymed Laboratories INC, San Francisco, CA). After incubation with secondary antibodies, the slices were incubated with streptavidin conjugated with Alexa Fluor 488 (diluted 1:1000) (Cat#S32354, AB_2315383, Molecular Probes, Carlsbad, CA) for 2 h at 25 °C. IgG binding sites were detected by confocal laser scanning microscopy (LSM510 Axiovert200M; Carl Zeiss Meditec, Göttingen, Germany). 4′,6-Diamidino-2-phenylindole (DAPI) was used for nuclear staining.

For double immunofluorescence, cryosections of fixed specimens were incubated at room temperature with a mixture of two primary antibodies: rabbit anti-CYP46A1 polyclonal antibody (Cat#12486-1-AP, AB_2090661, Proteintech, Rosemont, IL) (1:100) and mouse anti-NeuN monoclonal antibody (Cat#ab104224, Abcam, Cambridge, MA) (1:200). Subsequent antibody detection was performed with a mixture of two secondary antibodies, FITC-conjugated goat anti-rabbit IgG (H + L) (Cat#Zymed81-6111, Zymed Laboratories Inc, South San Francisco, CA) (1:200) and Alexa Fluor594-conjugated goat anti-mouse IgG (Cat#A-11005, Thermo Fischer Scientific, Waltham, MA) (1:200). After several washes with PBS, colocalization of CYP46A1 and NeuN was observed under a confocal microscope.

For quantification of immunohistochemical data, images of each section (5 sections per animal) were captured. Digital images were analyzed, and the average intensity of immunofluorescence was measured using Image-Pro Plus software (Media Cybernetics, Rockville, MD).

### Cholesterol measurements

After pressure loading, the retina was detached from sclera. Total lipids were extracted from the retina according to Folch’s method with chloroform/methanol^54^, and quantified using Cholestest® (Sekisui Medical Corp. Tokyo, Japan).

### LC-MS/MS

#### 24(S)-HS

The measurement of 24(S)-HC was based on previously reported methods, with some modification^55^. Briefly, the retina of the empty eyecup was detached from sclera at the end of each experiment. After a rat retina was homogenized in distilled water, 24-hydroxy-cholesterol-d_7_ was added to the suspension as an internal standard. Butylated hydroxytoluene and 1 N potassium hydroxide were added to the suspension, and then saponified at 37 °C for 1 h. After saponification, distilled water was added, and 24S-hydroxycholesterol was extracted with hexane. The extract was evaporated to dryness, and the residue was picolynoyl-esterified and subjected to liquid chromatography-tandem mass spectrometry (LC-MS/MS). For measurement of 24(S)-HC in retina, an API-4000 triple stage quadrupole mass spectrometer equipped with positive electrospray ionization (ESI) (AB Sciex, Mass, USA) connected to Nexera ultra high performance liquid chromatography systems (Shimadzu, Kyoto, Japan) was employed. The column was a Hypersil GOLD column (150 × 2.1 mm, 3 μm, Thermo Fisher Scientific, MA, USA) used at 40 °C. The mobile phase consisting of 0.1% acetic acid (solvent A) and acetonitrile-methanol (50:50, v/v) (solvent B) was used with a gradient elution. For quantification of 24S-hydroxycholesterol, transition of *m/z* 635.4/512.0 and *m/z* 642.4/519.5 were selected for 24S-hydroxycholesterol and 24S-hydroxycholesterol-d_7_, respectively. Results were expressed as 24(S)-HC levels (ng) per total retinal weight (g).

#### AlloP

Three eyes were examined by LC-MS/MS in each condition. The measurement of AlloP was based on previously reported methods^30^.

### Light Microscopy

At the end of each experiment, eyecup preparations were fixed in 2.5% glutaraldehyde in 0.1 M phosphate buffer overnight at 4 °C. The fixed eyecups were rinsed in 0.1 M phosphate buffer and placed in 1% buffered osmium tetroxide for 60 minutes. The eyecups were dehydrated with an ethanol dilution series, embedded in epoxy resin (Epon 812, TAAB Laboratories, Aldermaston, UK) and cut into 1 μm thick semi-thin sections. The tissue was then stained with toluidine blue and evaluated by light microscopy.

### Data Analysis

In histological studies, we examined the middle portion of the retina, greater than 1,200 μm away from the center of the optic disc along the inner limiting membrane (ILM) according to previously described methods^13^,^14^. The nerve fiber layer thickness (NFLT) was measured by light microscopy along 5–6 lines perpendicular to the pigment epithelium at a distance of 15 μm from each other around 1,200 μm away from the center of the optic disc (Fig. S3-2). The average NFLT was determined in 10 different light micrographs taken from 3 to 5 eyecup samples in each condition, divided by total retinal thickness, and mean ± standard deviation (SD) was analyzed and compared with control.

The severity of neuronal damage was assessed by light microscopy in ten fields from each experiment using a neuronal damage score (NDS) as previously described^13^. The NDS was determined in 10 different light micrographs taken from 3 to 5 eyecup samples in each condition.

The density of degenerated cells in the GCL was determined by counting 10 fields of 250 μm length at 10 different locations in light micrographs taken from the block of the middle retinal part 950 to 1450 μm away from the center of the optic disc.

These morphometrical parameters were assessed by three raters, who remained unaware of the experimental condition. Upon completion of data assessment, significance of individual differences among raters was evaluated using five randomly selected samples in each morphometric parameter by one-way analysis of variance (one-way ANOVA) followed by a post-hoc test. There were no significant differences among the raters in any of the morphometric measurements.

### Preparation of whole mounted retinas

The anterior part of the eye was removed by making an incision along the entire limbus. After incubation in the closed pressure system, retinas from five eyes in each group were processed for immunostaining as “whole mounted” retinas. The retina was carefully detached from the eye by making cuts along the ora serrata and optic nerve. Whole retinas were then flat-mounted, pinned out in an acrylic plate with the RGC layer facing upward using stainless steel pins, and fixed in 4% paraformaldehyde-0.1 M phosphate buffer overnight at 4 °C. After the samples were fixed, the tissue was rinsed with PBS three times. To block nonspecific binding, the tissue was incubated in 2% BSA in PBS containing 0.5% Triton X-100. The whole mounted retinas were incubated in the rabbit anti-NeuN polyclonal antibody solution (Cat#ab104225, Abcam) (1:100) by gently shaking at 4 °C, overnight. After rinsing 3 times using PBS, the retina was incubated in FITC-conjugated secondary antibody (goat anti-rabbit IgG (H + L)) (Cat#81-6111, Zymed Laboratories Inc) (1:300). The retina tissue was then rinsed 3 times with PBS and mounted on glass slides using 50% PBS and 50% glycerol.

The retinal flat-mounts were imaged throughout the GCL in each of the four defined retinal quadrants 4 mm from the optic nerve head using a confocal microscope. Each quadrant was analyzed using a 1 mm^2^ frame, and counted using Image-Pro Plus software. The density of NeuN positive RGCs per square millimeter was averaged and compared in experimental and control retinas^56^. RGC counts were analyzed using Image-Pro Plus software.

### Apoptosis

To visualize apoptotic cells, we used the DeadEndTM Colorimetric TUNEL System (Promega, Madison, WI) to determine the apoptotic cells according to the manufacturer’s instructions. The nuclei were counterstained with DAPI. Five retinal sections were randomly selected per each condition. After the length of each section was measured (Image-Pro Plus software), the TUNEL-positive cells were counted in the whole section length (between ora serrata to ora serrata). The number of apoptotic cells was expressed per 200 *μ*m of retinal section^57^.

### Statistics

Data were double-checked and analyzed using the Statistical Package for Bioscience V9.53 (SPBS) (Nankodo Publisher, Tokyo, Japan) on a personal computer. Descriptive statistical results were presented using the mean values (mean) ± standard deviation (SD). If the data were normally distributed, *p* values were calculated by Student’s unpaired t-test. If the data were not consistent with normal distribution, *p* values were calculated by Wilcoxon-Mann-Whitney non-parametric test. For all analyses, *p* values were considered statistically significant, when the values were less than 0.05 (two-tailed).

## Additional Information

**How to cite this article**: Ishikawa, M. *et al*. 24(S)-Hydroxycholesterol protects the *ex vivo* rat retina from injury by elevated hydrostatic pressure. *Sci. Rep.* **6**, 33886; doi: 10.1038/srep33886 (2016).

## Supplementary Material

Supplementary Information

## Figures and Tables

**Table 1:** Effects of pressure elevation and administration of 1 μM 24(S)-HC or 10 μM voriconazole on the NFLT, NDS, and density of damaged cells in the GCL.

Condition	NFLT vs. RT (%) [*p value vs. 10 mmHg*]	NDS [*p*]	Damaged cells in the GCL [*p*]
10 mmHg	1.32 ± 0.69 [−]	0.2 ± 0.4 [−]	3.4 ± 1.0 [−]
35 mmHg	1.40 ± 0.91 [0.827]	0.2 ± 0.4 [01.708000]	3.1 ± 1.2 [0.551]
75 mmHg	10.3 ± 2.02 [p < 0.0001]*	0.6 ± 0.5 [0.08564]	11.6 ± 2.4 [p < 0.0001]*
10 mmHg + 1 μM 24(S)-HC	1.39 ± 0.87 [0.844]	0.2 ± 0.4 [0.7091.000]	3.2 ± 1.7 [0.752]
35 mmHg + 1 μM 24(S)-HC	1.28 ± 0.84 [0.909]	0.2 ± 0.4 [0.7091.000]	4.2 ± 2.4 [0.343]
75 mmHg + 1 μM 24(S)-HC	1.69 ± 0.79 [0.279]	0.4 ± 0.5 [0.31436]	4.9 ± 2.2 [0.065]
10 mmHg + 10 μM Voriconazole	1.51 ± 0.63 [0.557]	3.8 ± 0.4 [p < 0.0001]*	51.4 ± 10.3 [p < 0.0001]*
35 mmHg + 10 μM Voriconazole	1.41 ± 0.73 [0.710]	3.5 ± 0.5 [p < 0.0001]*	25.8 ± 6.7 [p < 0.0001]*
75 mmHg + 10 μM Voriconazole	1.67 ± 1.16 [0.423]	2.0 ± 0.7 [p < 0.0001]*	18.0 ± 4.7 [p < 0.0001]*

Data are mean ± SD. NFLT vs. RT (%) refers to the NFLT percentage of total RT. The density of damaged cells in the GCL was counted per 250 μm of retina. P values in NFLT vs. RT (%) and damaged cells in the GCL were calculated by Student’s unpaired t-test (*p < 0.0001), and those in NDS were by Wilcoxon-Mann-Whitney non-parametric test (*p < 0.0001).

**Table 2:** Effects of 10 μM voriconazole and 24(S)-HC (1 μM, 10 μM, 30 μM,) or glutamate receptor antagonists (MK801, GYKI, MK801 + GYKI) on the NFLT, NDS, and density of damaged cells in the GCL.

Condition	NFLT vs. RT (%) [*p value vs. 10 mmHg*]	NDS [P]	Damaged cells in GCL [P]
10 mmHg + 10 μM Voriconazole + 1 μM 24(S)-HC	1.57 ± 0.60 [0.397]	3.7 ± 0.5 [p < 0.0001]*	60.5 ± 15.2 [p < 0.0001]*
10 mmHg + 10 μM Voriconazole + 10 μM 24(S)-HC	1.02 ± 0.58 [0.191]	1.4 ± 0.5 [p < 0.0001]*	23.3 ± 6.4 [p < 0.0001]*
10 mmHg + 10 μM Voriconazole + 30 μM 24(S)-HC	1.62 ± 1.05 [0.460]	0.3 ± 0.5 [0.500]	4.9 ± 2.6 [0.106]
10 mmHg + 10 μM Voriconazole + 1 μM MK801	1.52 ± 1.36 [0.917]	3.7 ± 0.5 [p < 0.001]*	49.6 ± 13.0 [p < 0.0001]*
10 mmHg + 10 μM Voriconazole + 1 μM GYKI	0.75 ± 0.68 [0.079]	3.7 ± 0.5 [p < 0.001]*	39.4 ± 16.0 [p < 0.0001]*
10 mmHg + 10 μM Voriconazole + 1 μM MK801 + 1 μM GYKI	1.32 ± 0.82 [1.000]	0.5 ± 0.5 [0.175]	2.7 ± 2.0 [0.335]

Data are mean ± SD. NFLT vs. RT (%) refers to the NFLT percentage of total RT. The density of damaged cells in the GCL was counted per 250 μm of retina. P values in NFLT vs. RT (%) and damaged cells in the GCL were calculated by Student’s unpaired t-test (*p < 0.0001), and those in NDS were by Wilcoxon-Mann-Whitney non-parametric test (*p < 0.0001).
