# Supplementary material for: 24(S)-Hydroxycholesterol protects the ex vivo rat retina from injury by elevated hydrostatic pressure
Source: Sci Rep. 2016 Sep 22;6:33886. doi: 10.1038/srep33886 (PMC5032171; doi:10.1038/srep33886)
Supplement: Supplementary Information [file srep33886-s1.doc]

**Supplementary information**

Title: 24(S)-Hydroxycholesterol protects the *ex vivo* rat retina from injury

by elevated hydrostatic pressure

Authors List:

^1^Makoto Ishikawa, ^1^Takeshi Yoshitomi, ^2,3,4^Charles F. Zorumski,

and ^2,3,4^Yukitoshi Izumi

^1^Department of Ophthalmology,

Akita University Graduate School of Medicine, Akita 010-8543, Japan

&

^2^the Taylor Family Institute for Innovative Psychiatric Research,

^3^Center for Brain Research in Mood Disorders, ^4^Department of Psychiatry,

Washington University School of Medicine, St. Louis, M.O, USA.

**Supporting Informations of Figures and tables.**

**Supporting data of Fig. 1B. Validation of internal control**

We assessed the validation by *rps16*^1^, *gapdh*^2^, and *actb*^3^, which were used as housekeeping genes in previous study using glaucoma animal models. **Table S1B-1** and **S1B-2** show the gene expression profiling of these housekeeping genes at each pressure using Multiplate RG soft (Takara Bio Inc., Shiga, Japan). The expression of each of the internal controls at 10 mmHg was compared with pressure-loaded samples (35 mmHg or 75 mmHg) and statistically analyzed with the paired *t* test^4^ or Wilcoxon Signed-Rank Test.

The relationship between *rps16* and other internal control genes was statistically significant at 35 mmHg using paired *t* test^4^ or Wilcoxon Signed-Rank Test (**Table S1B-1**). At 75 mmHg, the relationship between *rps16* and *actb* was statistically significant by paired *t* test (p<0.03) and Wilcoxon Signed-Rank Test (p<0.05). The relationship between *rps16* and *gapdh* was also statistically significant using paired *t* test, while there seemed no significant difference between *rps16* and *gapdh by* Wilcoxon Signed-Rank Test (**Table S1B-2**). Taken together, we selected *rps16* as the internal control, which resulted more stable.

**Table S1B-1**. Comparison of expression profile of house keeping genes *rps16*, *gapdh*, and *actb* for the quantitative real time PCR at 10 mmHg and 35 mmHg.

|  | *rps16* (n=10) | *gapdh* (n=10) | *actb* (n=10) |
| --- | --- | --- | --- |
| Mean value | 1.21±0.20 | 1.74±0.65 | 1.33±0.16 |
| Maximum | 1.50 | 3.00 | 1.50 |
| Minimum | 1.00 | 0.91 | 1.00 |
| *p (paired t-test)* | vs. *rps*16 | 0.04 | 0.03 |
| *p (Wilcoxon Signed-Rank Test)* | vs. *rps*16 | p<0.05 | p<0.05 |

**Table S1B-2.** Comparison of expression profile of house keeping genes *rps16*, *gapdh*, and *actb* for the quantitative real time PCR at 10 mmHg and 75 mmHg.

|  | *rps16* (n=10) | *gapdh* (n=10) | *actb* (n=10) |
| --- | --- | --- | --- |
| Mean value | 1.09±0.16 | 1.35±0.31 | 1.37±0.36 |
| Maximum | 1.3 | 1.75 | 1.80 |
| Minimum | 0.9 | 0.92 | 0.90 |
| *p* | vs. *rps*16 | 0.04 | 0.03 |
| *p (Wilcoxon Signed-Rank Test)* | vs. *rps*16 | n.p | p<0.05 |

**References**

1. Mac Nair CE, Schlamp CL, Montgomery AD, Shestopalov VI, Nickells RW. Retinal glial responses to optic nerve crush are attenuated in Bax-deficient mice and modulated by purinergic signaling pathways. J Neuroinflammation. 2016 Apr 28;13(1):93.
2. Bogner B, Schroedl F, Trost A, Kaser-Eichberger A, Runge C, Strohmaier C, Motloch KA, Bruckner D, Hauser-Kronberger C, Bauer HC, Reitsamer HA. Aquaporin expression and localization in the rabbit eye. Exp Eye Res. 2016 Jun;147:20-30.
3. Liu X, Huang P, Wang J, Yang Z, Huang S, Luo X, Qi J, Shen X, Zhong Y. The Effect of A2A Receptor Antagonist on Microglial Activation in Experimental Glaucoma.Invest Ophthalmol Vis Sci. 2016 Mar 1;57(3):776-86.
4. Hagihara K, Nishikawa T, Isobe T, Song J, Sugamata Y, Yoshizaki K. IL-6 plays a critical role in the synergistic induction of human serum amyloid A (SAA) gene when stimulated with proinflammatory cytokines as analyzed with an SAA isoform real-time quantitative RT-PCR assay system. Biochem Biophys Res Commun. 314 (2004) 363–369

**Primer list**

*rps16*

GenBank accession number NM_001169146.1

Forward (F) and reverse (R) primer sequences

F: GAAATGGGCTCATCAAGGTGAA

R: ACGGACCCGGATATCCACA

Product size (bp) 131

*gapdh*

GenBank accession number NM_017008.4

Forward (F) and reverse (R) primer sequences

F: GGCACAGTCAAGGCTGAGAATG

R: ATGGTGGTGAAGACGCCAGTA

Product size (bp) 143

*actb*

GenBank accession number NM_031144.3

Forward (F) and reverse (R) primer sequences

F: GGAGATTACTGCCCTGGCTCCTA

R: GACTCATCGTACTCCTGCTTGCTG

Product size (bp) 150

**Table. S1B-3. Real time -PCR analysis of *cyp46a1* mRNA.**

| Experiment | **10 mmHg** | **35 mmHg** | **75 mmHg** |
| --- | --- | --- | --- |
| 1 | 0.94 | 1.8 | 2.89 |
| 2 | 1.1 | 1.3 | 2.28 |
| 3 | 0.8 | 1.9 | 2.54 |
| 4 | 0.86 | 2 | 2.29 |
| 5 | 1 | 1.81 | 2.67 |
| 6 | 1.11 | 1.86 | 2.29 |
| Average | 0.97 | 1.78 | 2.49 |
| SD | 0.13 | 0.25 | 0.25 |
| Wilcoxon-Mann-Whitney non-parametric test*(p)* | vs. 10mmHg | p<0.0001 | p<0.0001 |

**Table. S1C.** ELISA analysis of CYP46A1.

|  |  | **10 mmHg** | **35 mmHg** | **75 mmHg** |
| --- | --- | --- | --- | --- |
| Exper-iment | Total number of retina | **8** | **8** | **8** |
| 1 | CYP46A1 ng/mg protein (Number of retina) | 56.02 (2) | 65.52 (2) | 77.81 (2) |
| 2 |  | 50.12 (2) | 70.23 (2) | 75.59 (2) |
| 3 |  | 45.98 (2) | 66.22 (2) | 81.01 (2) |
| 4 |  | 55.12 (2) | 58.91 (2) | 85.92 (2) |
|  | Average | 51.81 | 65.22 | 80.08 |
|  | SD | 4.67 | 4.69 | 4.48 |
|  | Wilcoxon-Mann-Whitney non-parametric test (*p*) | vs. 10 mmHg | p<0.0001 | p<0.0001 |

**Table. S1G.** Quantification of immunofluorescence induced by anti-CYP46A1 antibody.

| Experiment | **10 mmHg** | **35 mmHg** | **75 mmHg** |
| --- | --- | --- | --- |
| 1 | 12 | 25 | 59 |
| 2 | 16 | 21 | 57 |
| 3 | 20 | 22 | 48 |
| 4 | 3 | 28 | 50 |
| 5 | 18 | 20 | 55 |
| Average | 13.8 | 23.2 | 53.8 |
| SD | 6.7 | 3.3 | 4.7 |
| Wilcoxon-Mann-Whitney non-parametric test (*p*) | vs.  10 mmHg | *p*=0.028 | *p*<0.0001 |

**Table. S2B**. Total cholesterol measurement at each pressure.

| (mg/g) | |  |  |
| --- | --- | --- | --- |
| Experiment | **10 mmHg** | **35 mmHg** | **75 mmHg** |
| 1 | 2.1 | 2 | 1.8 |
| 2 | 2.2 | 1.9 | 1.7 |
| 3 | 2.2 | 2 | 1.75 |
| 4 | 2 | 2 | 1.8 |
| 5 | 2 | 2.1 | 1.9 |
| 6 | 2.1 | 2 | 1.8 |
| 7 | 2.1 | 1.9 | 1.7 |
| 8 | 2.2 | 2 | 1.6 |
| 9 | 2.1 | 2 | 1.7 |
| 10 | 2 | 2 | 1.8 |
| Average | 2.1 | 1.99 | 1.755 |
| SD | 0.08 | 0.06 | 0.08 |
| Wilcoxon-Mann-Whitney non-parametric test *(p)* | vs. 10 mmHg | 0.004 | *p*<0.001 |

**Table. S2C.** LC-MS/MS analysis of 24(S)-HC

(ng/g protein).

| aCSF | **10 mmHg** | **35 mmHg** | **75 mmHg** |
| --- | --- | --- | --- |
| 1 | 1992 | 2292 | 2368 |
| 2 | 2026 | 2128 | 2487 |
| 3 | 1800 | 2134 | 2619 |
|  |  |  |  |
| Average | 1939.3 | 2184.7 | 2491.3 |
| SD | 121.9 | 93.0 | 125.6 |
| Wilcoxon-Mann-Whitney non-parametric test *p* | vs. 10 mmHg | *p*<0.05 | *p*<0.05 |

**Table. S2D-1.** LC-MS/MS analysis of 24(S)-HC without voriconazole.

(ng/g retina)

| aCSF | 10 mmHg | 35 mmHg | 75 mmHg |
| --- | --- | --- | --- |
| 1 | 676.2 | 782.9 |  |
| 2 |  | 733.9 | 1068.3 |
| 3 | 704.7 | 882.9 | 857.7 |
| 4 | 586.9 | 746.5 | 843.3 |
| Average | 655.9  61.5 | 786.6  67.5 | 923.1  126.0 |
| SD |
| Wilcoxon-Mann-Whitney non-parametric test *p* | vs 10 mmHg | *p*=0.046 | *p*=0.029 |

**Table. S2D-2.** LC-MS/MS analysis of 24(S)-HC with 1 μM voriconazole.

(ng/g retina)

| 1 uM Vor | 10 mmHg | 35 mmHg | 75 mmHg |
| --- | --- | --- | --- |
| 1 | 501.0 | 590.2 | 648.8 |
| 2 | 446.6 | 669.8 | 799.3 |
| 3 | 500.6 | 784.8 | 742.0 |
| Average | 482.7  31.3 | 681.6  97.8 | 730.0  76.0 |
| SD |
| Wilcoxon-Mann-Whitney non-parametric test *(p*) (vs. aCSF) | 0.06  (10 mmHg) | 0.89  (35 mmHg) | 0.07  (75 mmHg) |
|  |  |  |  |

| **Table. S2D-3.** LC-MS/MS analysis of 24(S)-HC with 10 μM　voriconazole.  (ng/g retina) | | | |
| --- | --- | --- | --- |
| 10 uM Vori | 10 mmHg | 35 mmHg | 75 mmHg |
| 1 | 441.2 | 391.8 | 395.8 |
| 2 | 408.4 | 389.4 | 456.3 |
| 3 | 496.6 | 590.7 | 445 |
| Average | 457.3  44.6 | 457.3  115.5 | 432.4  32.2 |
| SD |
| Wilcoxon-Mann-Whitney non-parametric test *(p*) vs. aCSF *(p)* | *p*=0.009  (10 mmHg) | *p*=0.011  (35 mmHg) | *p*=0.003  (75 mmHg) |

**Table. S2E.** LC-MS/MS analysis of AlloP in the retina treated without 10 μM voriconazole.

**(ng/g retina)**

| Vori 10 uM | 10 mmHg | 35 mmHg | 75 mmHg |
| --- | --- | --- | --- |
| 1 | 1.45 | 32.7 | 62.43 |
| 2 | 1.61 | 62.4 | 62.15 |
| 3 | 2.23 | 55.8 | 55.64 |
|  |  |  | 63.97 |
| Average | 1.80  1.13 | 50.3  15.60 | 61.0  4.38 |
| SD |
| Wilcoxon-Mann-Whitney non-parametric test *(p*) | vs 10 mmHg | *p*=0.006 | *p*<0.001 |

**Fig. S2F.** LC-MS/MS analysis of AlloP in the retina treated without voriconazole.

(ng/g retina)

| Vori 10 uM | 10 mmHg | 35 mmHg | 75 mmHg |
| --- | --- | --- | --- |
| 1 | 1.61 | 5.9 | 73.90 |
| 2 | 1.53 | 5.13 | 55.50 |
| 3 | 2.20 | 1.61 | 54.00 |
| Average | 1.78 | 4.21 | 61.13 |
| SD | 0.94 | 2.82 | 11.08 |
| Wilcoxon-Mann-Whitney non-parametric test *(p*) | vs 10 mmHg | 0.150 | *p*<0.001 |

**Fig. S3-1**. Structural changes of retinal ganglion cells and their axons induced by pressure elevation.


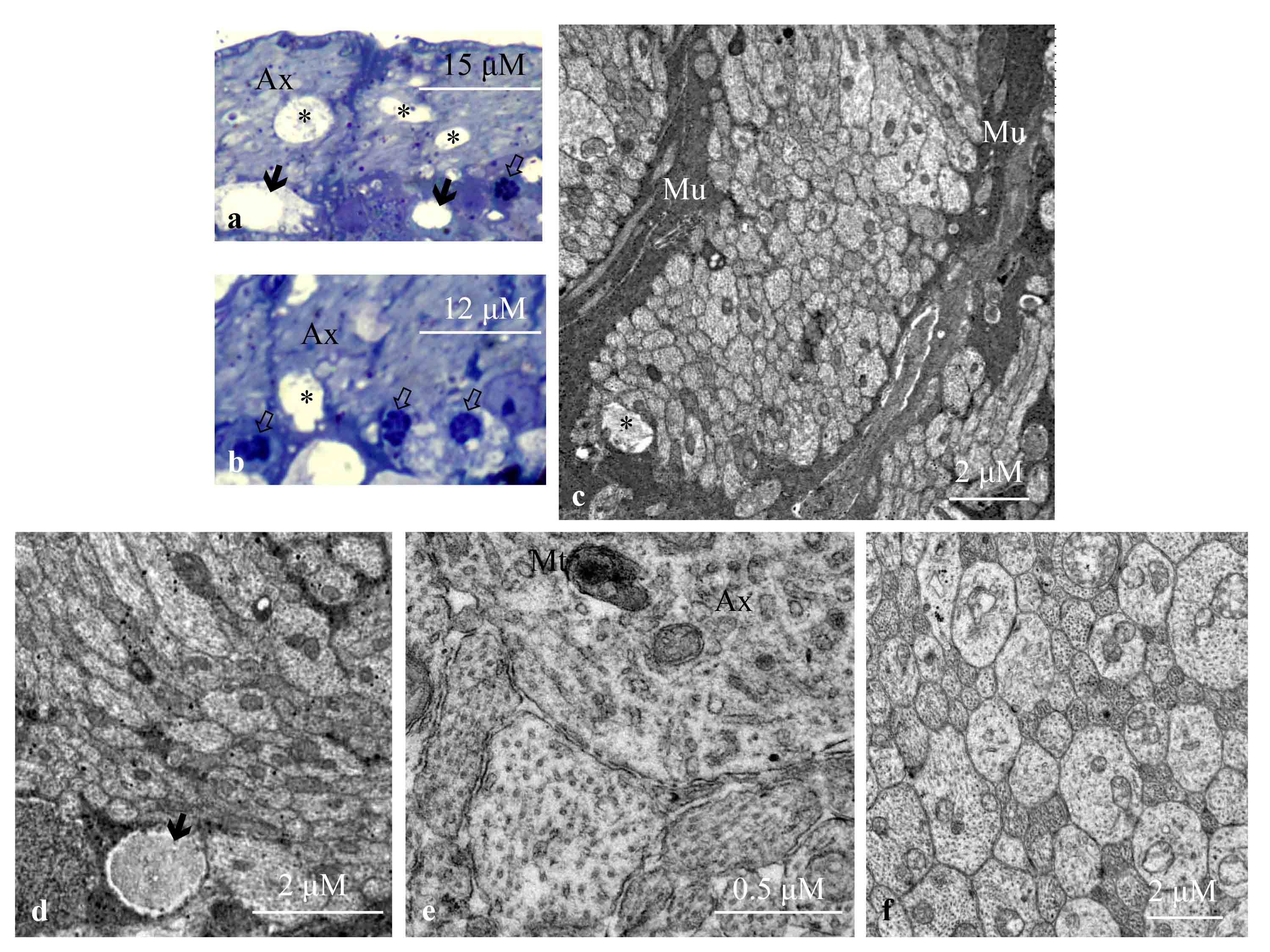


**a and b.** Light micrographs of the NFL and GCL in the middle retinal segments. Axonal swelling (Ax) of the RGCs was observed after exposure to elevated hydrostatic pressure (75 mm Hg). Note the vacuoles in the NFL (*). Arrows indicate the degeneration of the RGCs. A number of RGCs showed nuclear pyknosis (open arrows). **c–f.** Electron micrographs of the NFL. **c and d.** After exposure to high pressure (75 mm Hg) for 24 hours, numerous axons of various sizes in the NFL were swollen. Asterisk and arrow indicate the vacuole and axonal degeneration, respectively. Mu, cytoplasm of the Müller cell. **e.** High magnification of the swollen axon (Ax). Note the degenerated mitochondria (Mt) and disarrangement of filamentous structures in the swollen axons. **f.** Control NFLs in the middle retinal segments incubated at 10 mmHg. The NFL contained numerous axons of various sizes. Each axon contained regularly arranged neurofilaments and microtubules.

**Fig. S3-2.** NFLT measurement.


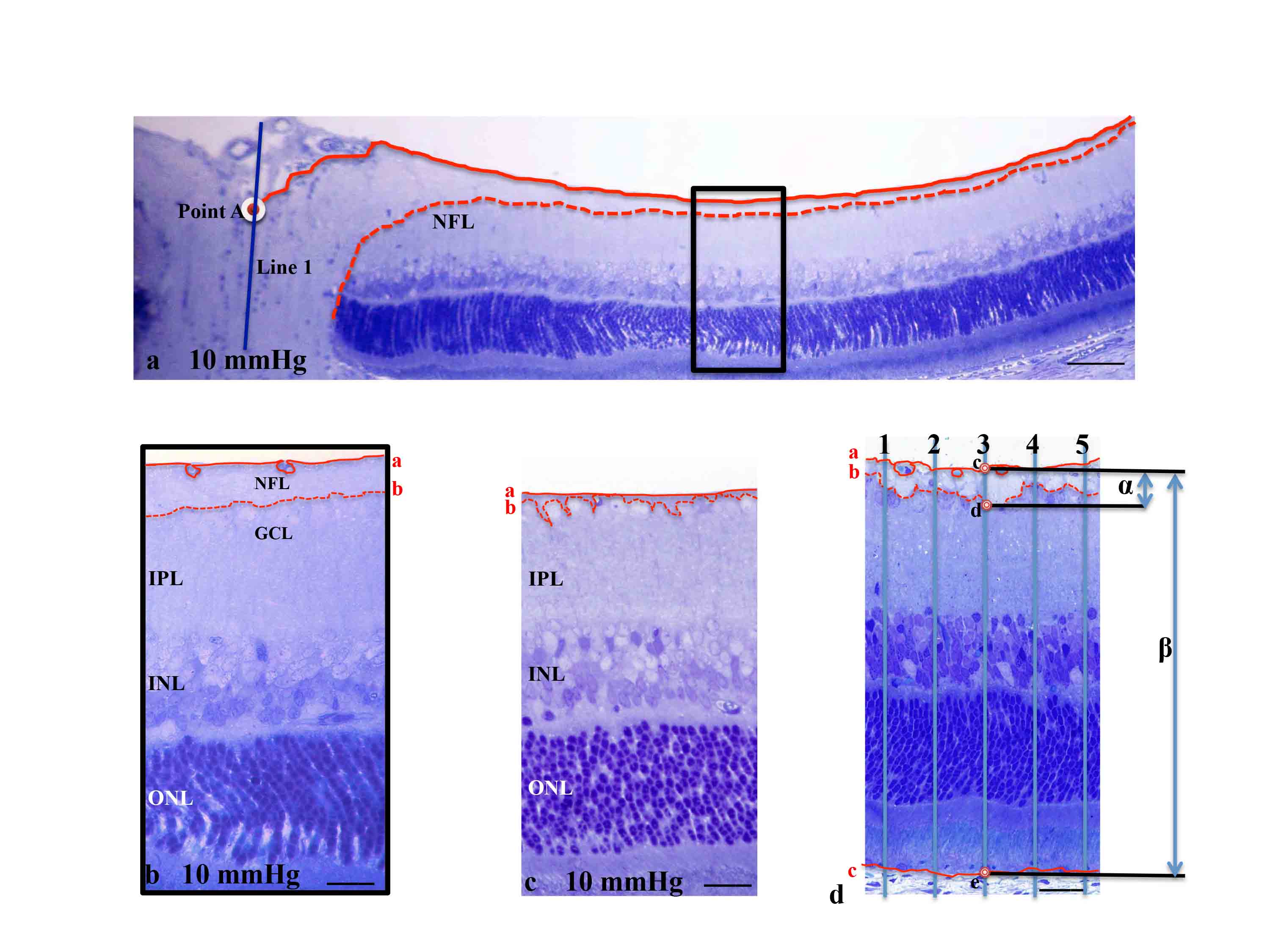


**(a)** Light micrograph of the posterior region of the eyecup preparation incubated at 10 mmHg. The NFL was traced as a layer between the ILM and the GCL. Solid line: the ILM; dashed line: posterior boundary of the NFL. We constructed a perpendicular line (**line 1**) through the center of the optic disc, and the distance was measured along the ILM from the crossing point (**point A**; white circle) of the ILM and line 1. It is easy to identify the NFL by light microscopy within the retinal region 1000 μm proximal to **point A**.

**(b)** High magnification of the rectangular area depicted in **(a)**. Line a traces the ILM and the posterior border of the retinal vessels. The prominent NFL can be easily traced in this region, but is gradually diminished in thickness, especially in the retinal region 1200 μm distal from point A. In the present study, we examined the NFL thickness of the retinal region greater than 1200 μm away from **point A**.

**(c, d)** The NFL of retinas incubated at 10 and 75 mm Hg. The lines are as described in **(a)**. The NFL was defined as a layer between the ILM and the GCL. The NFL and the pigment epithelial layer were traced. The six lines perpendicular to the pigment epithelial layer were constructed at a distance of 15 μm from each other, and the crossing points with the ILM (**line a**), the posterior boundary of the ILM (**line b**), and the pigment epithelial layer (**line c**) are marked as points c, d, and e, respectively. The distance between points c and d is defined as the NFLT (**α**), and the distance between points c and e as total RT (**β**). The percentage of NFLT is measured as **α/β** ×100 (%).

(This figure plate is a copy of Fig. 2 in article entitled “Downregulation of glutamine synthetase via GLAST suppression induces retinal axonal swelling in a rat *ex vivo* hydrostatic pressure model” by Ishikawa et al. Invest Ophthalmol Vis Sci. 2011;52:6604–6616) DOI:10.1167/iovs.11-7375. We fully acknowledge for the Association for Research in Vision and Ophthalmology as the copyright holder.)

**Fig. S3-3.** Effects of 1 μM voriconazole on retinal morphology at each pressure.

**
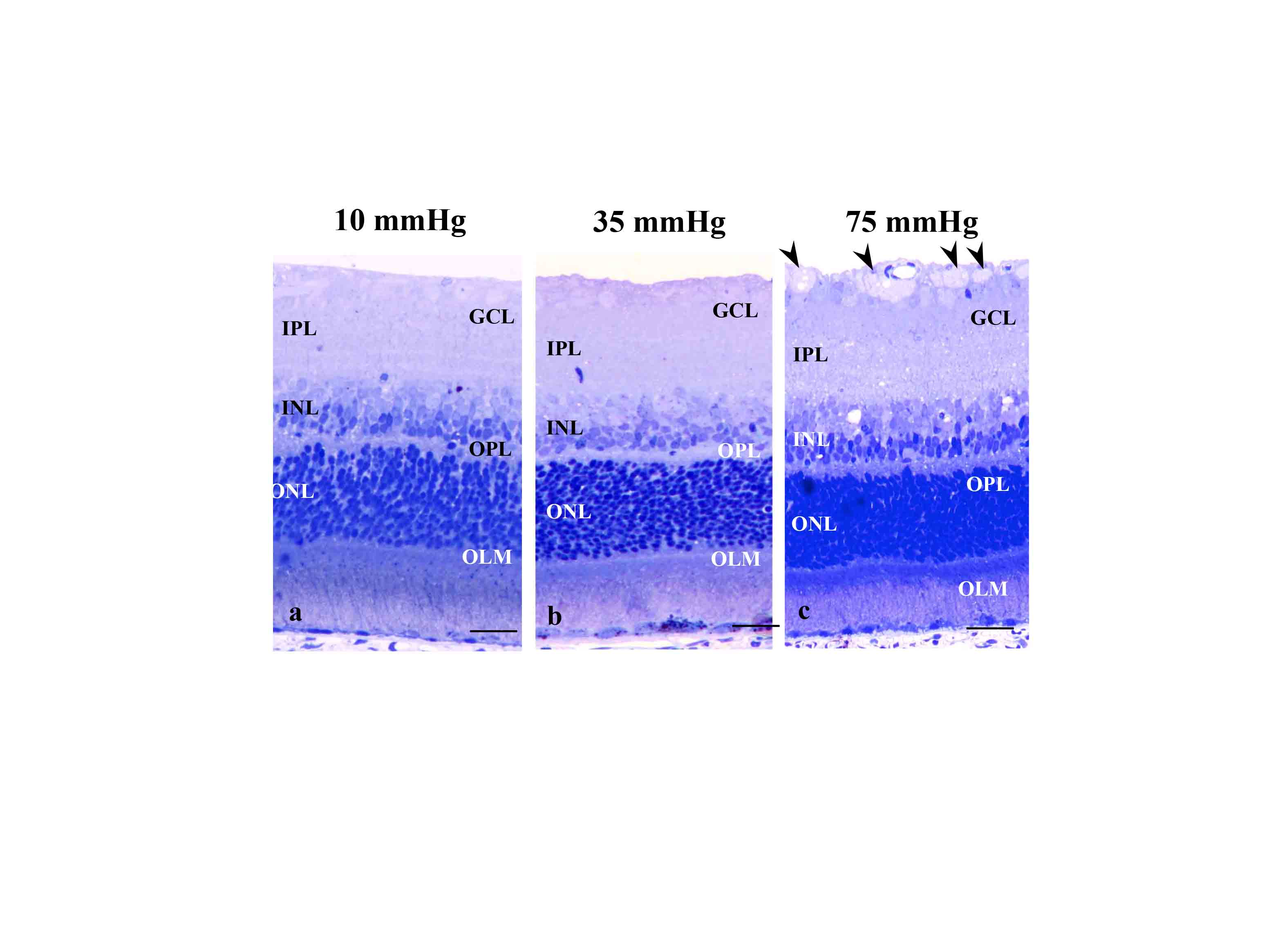
**

Light micrograph of the posterior region of the eyecup preparation incubated with 1 μM voriconazole at each pressure. Administration of 1 μM voriconazole showed no remarkable changes. Arrowheads indicate axonal swelling induced by pressure elevation (75 mmHg).

**Table. S5F and S5G. Density of NeuN-positive using RGCs in the whole mount retina.**

|  | 10 mmHg | 75 mmHg | 75mmHg+ 24(S)-HC | 10 mmHg+ Vor | 10 mmHg+Vor+ 24(S)-HC |
| --- | --- | --- | --- | --- | --- |
| 1 | 2512 | 1737 | 2655 | 1456 | 2677 |
| 2 | 2601 | 1823 | 2314 | 1698 | 2312 |
| 3 | 2921 | 1933 | 2433 | 1697 | 2448 |
| 4 | 2445 | 2034 | 2509 | 1526 | 2556 |
| 5 | 2433 | 1889 | 2684 | 1589 | 2135 |
| Average | 2582.4  201 | 1883.2  112 | 2519  154 | 1593.2  106 | 2425.6  211 |
| SD |
| *p* | - | vs.10mmHg p=0.004 | vs.75mmHg p=0.004 | - | vs.10 mmHg+ Vor p=0.004 |

Data was analyzed using Wilcoxon-Mann-Whitney non-parametric test*.*

**Fig S6D. Density of apoptotic cells in retina/200 μm.**

|  | 10 mmHg | 75 mmHg | 75mmHg+  24(S)-HC |
| --- | --- | --- | --- |
| 1 | 1 | 35 | 7 |
| 2 | 2 | 37 | 8 |
| 3 | 3 | 42 | 12 |
| 4 | 0 | 40 | 8 |
| 5 | 2 | 38 | 9 |
| Average | 1.6  1.1 | 38.4  2.7 | 8.8  1.9 |
| SD |
| Wilcoxon-Mann-Whitney non-parametric test*.(p)* | - | p=0.004  vs.10 mmHg | p=0.004  vs.75 mmHg |

Data was analyzed using Wilcoxon-Mann-Whitney non-parametric test*.*

**Table 1 Source data.**

**Table 2 Source data.**
